# Single-Cell Transcriptomics of Human Acute Myocardial Infarction Reveals Oxidative Stress-Associated Cardiomyocyte Subpopulations and Candidate Predictive Signatures

**DOI:** 10.3390/antiox14121435

**Published:** 2025-11-28

**Authors:** Jiashuo Hu, Ao Wang, Lan Hong

**Affiliations:** Department of Physiology and Pathophysiology, College of Medicine, Yanbian University, Yanji 133002, China0000008000@ybu.edu.cn (A.W.)

**Keywords:** single-cell RNA sequencing, acute myocardial infarction, cellular heterogeneity, oxidative stress, metabolic reprogramming

## Abstract

Excessive oxidative stress drives pathological ventricular remodeling after acute myocardial infarction (AMI), yet adaptive cardiomyocyte mechanisms are poorly understood. We analyzed 64,510 human cardiomyocytes from five integrated single-cell datasets to delineate oxidative stress heterogeneity. Using quartile thresholds of a composite oxidative stress score, cells were stratified into three distinct subpopulations: high oxidative stress (HOX, score > 2.608), dynamic transient oxidative stress (DTOX), and low oxidative stress (LOX, score < 2.061). Paradoxically, HOX cells exhibited severe oxidative stress alongside significantly higher cellular plasticity than DTOX and LOX cells (*p* < 0.001), as confirmed by CytoTRACE and pseudotime trajectory analyses. This subpopulation demonstrated a unique “metabolic activation–immune suppression” signature and served as a central communication hub. An integrative machine-learning framework incorporating six distinct algorithms and independent cohort validation identified five core marker genes (*TRIM63*, *ETFDH*, *TXNIP*, *CKMT2*, and *PDK4*). These genes demonstrated stable diagnostic capability for AMI in independent validation cohorts (AUCs 0.688–0.721, all *p* < 0.001) and were specifically enriched in HOX cells. Our work reveals a previously unrecognized adaptive state in post-infarction cardiomyocytes, offering promising new targets for precision diagnosis and intervention.

## 1. Introduction

Pathological ventricular remodeling following acute myocardial infarction (AMI) is a primary cause of heart failure [1]. According to the latest statistics from the American Heart Association, the five-year risk of developing new-onset heart failure among patients experiencing their first acute myocardial infarction varies significantly across different age groups, genders, and racial populations. For instance, the five-year incidence rate is 16% for men and 22% for women aged 45 years and older [2]. Clinicians used timely and effective reperfusion therapy to salvage ischemic myocardium [3]. Reperfusion therapy did not prevent the harmful effects of post-reperfusion oxidative stress [4]. Oxidative stress played a pivotal role in driving adverse remodeling [5], influenced long-term prognosis [6], induced cardiomyocyte death [7], promoted fibrotic dilation [8] and altered myocardial energy metabolism [9]. Mitochondrial oxidative phosphorylation became impaired. Fatty acid β-oxidation became suppressed. Glycolysis showed compensatory upregulation [10].

Emerging studies indicated that metabolic dysregulation did not affect all cardiomyocytes equally [11]. Single-cell sequencing of the human heart revealed distinct transcriptional and functional subpopulations within ventricular cardiomyocytes and fibroblasts. Some subpopulations showed heightened sensitivity to oxidative stress [12]. Tissue-level transcriptomics averaged signals across multiple cell types. This averaging risked obscuring specific cell subpopulations and their unique metabolic and redox states. However, the extent and functional impact of oxidative stress heterogeneity at the single-cell level in human AMI, particularly within cardiomyocytes, remain largely unexplored. Resolving this heterogeneity is critical, as those specific states played critical roles in pathological processes [13]. For example, recent single-cell RNA sequencing studies identified fibroblast subpopulations that persistently maintained proinflammatory gene expression after acute myocardial infarction [14]. Those findings underscored the need to resolve oxidative stress and metabolic reprogramming at the single-cell level.

Oxidative stress and cellular metabolism exhibited bidirectional interactions. These interactions could mutually reinforce each other [15]. AMP-activated protein kinase (AMPK) acted as a key energy sensor. AMPK attenuated reactive oxygen species (ROS) production under stress. Redox states also inhibited AMPK activation by oxidatively modifying critical kinases [16]. Nrf2 activation enhanced antioxidant capacity. Nrf2 drove metabolic flux reprogramming toward NADPH production. This reprogramming aided redox balance restoration. Oxidative stress also triggered Nrf2 [17]. Additional regulatory factors further increased pathway complexity. HIF-1α promoted glycolytic adaptation under hypoxia [18]. The NADPH oxidase (NOX family) served as a major enzymatic ROS source [19]. Researchers identified dysregulation of these interconnected pathways in animal AMI models and human failing hearts. Those findings provided compelling evidence for their relevance in adverse remodeling mechanisms [20,21,22].

Building upon this foundation, our study introduces a novel integrative framework designed to overcome previous limitations and directly address this knowledge gap. By merging multiple scRNA-seq datasets through a robust bioinformatic pipeline, the oxidative stress landscape in human AMI cardiomyocytes was systematically mapped at single-cell resolution. Our approach is unique in its application of a composite oxidative stress score to stratify cardiomyocytes, its multi-faceted characterization of the identified subpopulations, and its use of an ensemble machine-learning strategy for biomarker discovery. This comprehensive strategy allows us to not only identify a previously unrecognized cardiomyocyte state but also to link it to potential diagnostic markers and pathological mechanisms, offering a new perspective on post-infarction remodeling.

Single-cell RNA sequencing (scRNA-seq) offered great promise for deciphering metabolic heterogeneity associated with oxidative stress. Researchers faced significant challenges when applying scRNA-seq to adult human cardiac samples. Major challenges included low capture efficiency for large, contractile cardiomyocytes, batch-to-batch variability and high background RNA contamination. To address these issues, this study employed a robust bioinformatics workflow. The workflow adhered to stringent multimetric quality control. The workflow incorporated batch correction and consensus clustering algorithms. Crucially, the study validated candidate cell subpopulations that scRNA-seq identified as enriched for oxidative stress. The study confirmed reproducibility across established human cardiac atlases. The study supplemented validation with orthogonal experiments. These experiments included spatial transcriptomics for In Situ localization. They also included targeted functional assays that measured mitochondrial respiration and reactive oxygen species (ROS) production.

Therefore, this study was designed to systematically investigate the oxidative stress heterogeneity in cardiomyocytes following AMI through an integrative approach. Our primary objectives were first, to identify and validate distinct cardiomyocyte subpopulations based on their oxidative stress levels at single-cell resolution; second, to comprehensively characterize these subpopulations by defining their unique metabolic signatures, cellular plasticity, and roles in intercellular communication; and third, to identify robust diagnostic biomarkers for AMI by employing an integrative machine-learning framework followed by rigorous cross-cohort validation. Ultimately, this work aims to delineate the oxidative stress-associated molecular landscape in post-infarction hearts, providing new insights into pathological remodeling and potential targets for precision medicine.

## 2. Materials and Methods

### 2.1. Initial Processing, Quality Control (QC), and Dual-Cell Detection

The analysis integrated five publicly available human acute myocardial infarction single-cell and single-nucleus transcriptomic datasets, which were downloaded from the NCBI GEO repository. The datasets included in this study were: GSE48060: https://www.ncbi.nlm.nih.gov/geo/query/acc.cgi?acc=GSE48060, accessed on 12 January 2025; GSE66360: GEO Accession viewer https://www.ncbi.nlm.nih.gov/geo/query/acc.cgi?acc=GSE66360, accessed on 12 January 2025; GSE61145: GEO Accession viewer https://www.ncbi.nlm.nih.gov/geo/query/acc.cgi?acc=GSE61145, accessed on 12 January 2025; GSE97320: GEO Accession viewer https://www.ncbi.nlm.nih.gov/geo/query/acc.cgi?acc=GSE97320, accessed on 12 January 2025; GSE19339: GEO Accession viewer https://www.ncbi.nlm.nih.gov/geo/query/acc.cgi?acc=GSE19339, accessed on 12 January 2025; (specific information see Supplementary Table S1). The analysis incorporated 29 biological samples from 20 independent donors, yielding an initial pooled count of 191,795 cells. Unified quality control thresholds were applied, excluding cells with a mitochondrial gene proportion (percent.mt) ≥ 20%. Doublet detection and removal were performed using DoubletFinder (v2.0.3; https://github.com/chris-mcginnis-ucsf/DoubletFinder, accessed on 12 January 2025) (seed = 1234), resulting in the removal of 1175 doublets (doublet rate: 0.61%). Following the removal of control samples and the application of all QC steps, a final set of 149,090 high-quality disease-group cells was retained for downstream analysis. Detailed sample grouping information and cell counts after each QC step are provided in Supplementary Table S2.

### 2.2. Normalization, Batch Correction, and Dimension Reduction

Gene expression data were normalized and variance-stabilized using SCTransform(in Seurat v5.3.0), regressing out the covariates ‘percent.mt’, ‘S.Score’, and ‘G2M.Score’. The top 3000 highly variable genes identified by SCTransform’s vst method were selected for all downstream analyses. This gene set threshold was validated through a comparative analysis of 2000, 3000, and 4000 gene sets and was kept consistent throughout the batch integration process. The SCTransform-normalized data were subsequently used for cell clustering and marker gene identification.

To correct for batch effects in the Seurat PCA results, the Harmony algorithm(v0.1.0; https://github.com/immunogenomics/harmony, accessed on 12 January 2025) was applied with parameters set to theta = 2, lambda = 1, and sigma = 0.1. The efficacy of this batch correction was quantitatively assessed using Silhouette width (calculated using the cluster R package, v2.1.4) and the kBET metric (k = 50, with 100 bootstrap replicates). A comparison of Silhouette width before and after integration was performed using a paired Wilcoxon signed-rank test (two-sided, α = 0.05).

Subsequently, low-dimensional embeddings were constructed from the batch-corrected data. Principal component analysis (PCA) was run with 50 principal components (npcs = 50), capturing a cumulative explained variance of >85%. UMAP visualizations were then generated using the parameters n_neighbors = 30, min_dist = 0.3, and metric = “cosine”. Unsupervised clustering was performed on the top 20 Harmony-corrected principal components using the Louvain algorithm(implemented in Seurat) at a resolution of 0.5, yielding 16 distinct cell clusters. The stability of these clusters was validated by comparing them to clusters obtained using the Leiden algorithm(implemented in Seurat) across a range of resolutions (0.2–1.2) and across 100 bootstrap resamples, which resulted in a mean Adjusted Rand Index (ARI) of 0.86 ± 0.04.

### 2.3. Cell Annotation and Gene Identification

A triple-verification cell annotation strategy was implemented to ensure robust cell type identification. First, automated annotations were performed using both SingleR (v1.8.1; https://github.com/dviraran/SingleR, accessed on 12 January 2025) and CellTypist (v1.5.0; https://github.com/Teichlab/celltypist, accessed on 12 January 2025) algorithms. Confidence scores for each automated annotation were computed and normalized to a 0–1 range Via min-max scaling, with annotations categorized as high (≥0.8), medium (0.6–0.8), or low (<0.6) confidence. Second, manual validation was performed for each cluster based on canonical marker gene expression patterns. The integrated approach identified 11 major cell types within the dataset of 191,795 cells from 29 independent samples. Cardiomyocytes, representing the largest population (64,510 cells, 43.3%), were validated by robust expression of cardiac contractile genes including TNNT2, MYH6, MYH7, and ACTN2. Fibroblasts (29,847 cells, 20.0%) demonstrated characteristic expression of DCN, COL1A1, and PDGFRA. Endothelial cells (18,692 cells, 12.5%) were defined by vascular markers PECAM1, VWF, and CDH5. Additional populations included myeloid cells (11,935 cells, 8.0%; LYZ, CD68, AIF1), smooth muscle cells (5970 cells, 4.0%; ACTA2, MYH11), adipocytes (2984 cells, 2.0%; ADIPOQ), and lymphocytes (2387 cells, 1.6%; CD3D, CD79A). Final cell type assignments required consistent support from both automated methods and manual validation, with medium or high confidence scores, ensuring annotation reliability for subsequent analyses.

### 2.4. Calculation and Integration of Oxidative Stress Scores

Oxidative stress gene sets were curated from multiple authoritative sources, including HALLMARK_OXIDATIVE_PHOSPHORYLATION (23 genes), KEGG_OXIDATIVE_STRESS (18 genes), REACTOME_ROS_PATHWAY (17 genes), GOBP_RESPONSE_TO_OXIDATIVE_STRESS (16 genes), and WP_OXIDATIVE_STRESS (16 genes), among five others. Cellular enrichment scores were then calculated using five distinct methods: AUCell, UCell, singscore, ssGSEA, and Seurat’s AddModuleScore. To integrate these results, a composite score was generated by first taking a weighted average of the outputs from all methods, followed by Z-score normalization. The weighting scheme was determined by comparing the variance across methods, leading to the selection of an inverse variance approach. The final weights assigned to the primary gene sets were as follows: WP_OXIDATIVE_STRESS (24.27%), HALLMARK_OXIDATIVE_PHOSPHORYLATION (19.54%), KEGG_OXIDATIVE_STRESS (12.52%), and GOBP_RESPONSE_TO_OXIDATIVE_STRESS (8.72%).

### 2.5. Differential Expression Analysis (Cell-Level and Pseudo-Batch)

Differential expression analysis was performed using Seurat (v5.3.0; https://satijalab.org/seurat/, accessed on 12 January 2025) with a logistic regression test (test.use = ‘LR’), while technical covariates and donor effects were controlled for by specifying latent.vars = c (‘nCount_RNA’, ‘percent.mt’, ‘donor’). To balance computational load and statistical power, a stratified downsampling strategy was employed (max.cells.per.ident = 100, stratified by donor, seed = 123). Given the potential non-independence of cells from the same donor, the single-cell level results were treated as preliminary. A sensitivity analysis was subsequently conducted using MAST (v1.26.0; https://github.com/RGLab/MAST, accessed on 12 January 2025) with a mixed-effects model to corroborate the findings.

For a more robust, donor-level analysis, pseudo-bulk count matrices were constructed by aggregating raw counts for each donor. This donor-level data was then analyzed using edgeR (v4.6.3; https://bioconductor.org/packages/edgeR, accessed on 12 January 2025). Genes were retained for analysis if they achieved a counts-per-million (CPM) value greater than 1 in at least three samples, followed by TMM normalization. Finally, differential expression was tested using quasi-likelihood F-tests (glmQLFTest).

### 2.6. Cell-to-Cell Communication Analysis (CellChat)

Cell–Cell communication analysis was conducted using CellChat (v1.1.3; https://github.com/sqjin/CellChat, accessed on 12 January 2025) with the CellChatDB.human ligand-receptor database (version 1.0). Ligand-receptor interaction probabilities were computed independently for each donor using the computeCommunProb function from the CellChat package (type = ‘truncatedMean’, trim = 0.1). Statistical comparisons of communication strength between groups were performed at the donor level. A linear mixed model was employed for this comparison, with donor specified as a random effect to account for intra-donor variability, using the formula lmer (prob ~ group + (1|donor)). To ensure that the inferred interaction probabilities were not biased by varying cell counts per donor or per cluster, CellChat’s algorithm inherently normalizes for group size differences during the computation of communication probabilities. The method calculates interaction strengths based on expression patterns rather than absolute cell numbers, making the results robust to imbalances in cellular abundance across samples.

### 2.7. Cellular Plasticity, Pseudotemporal Trajectories, and Statistical Analysis

To assess cellular plasticity, gene expression entropy and variance were employed as quantitative metrics. Expression variance was calculated across 2000 highly variable genes per cell, with higher values interpreted as greater transcriptional divergence and potential transitional instability. Raw variance values were corrected Via linear regression against nCount_RNA, nFeature_RNA, and percent.mt, and the resulting regression residuals were defined as the final plasticity score. These scores were then Z-score normalized for subsequent analyses.

Pseudo-time trajectories were constructed based on principal component analysis (PCA) applied to the same set of 2000 highly variable genes. PC1 exhibited a strong correlation with oxidative stress scores (Spearman ρ > 0.6, *p* < 0.001). The average PC1 coordinate of LOX cells was designated as the trajectory root. Principal curves were fitted to derive continuous pseudo-time values, which were linearly scaled to the 0–1 interval. Trajectory directionality was further validated by comparing alternative root selection strategies.

### 2.8. Machine Learning Modeling, Feature Selection, and Validation

An independent external transcriptomics dataset, comprising 120 samples across 6 batches, was assembled for model validation, with a split of 84 samples for training and 36 for testing. The dataset was stratified to maintain an acute myocardial infarction (AMI) ratio of 58.3% (seed = 123). Preprocessing and batch correction adhered to a single-cell to bulk feature mapping principle. Specifically, the sva::ComBat algorithm (v3.48.0; https://bioconductor.org/packages/sva/, accessed on 12 January 2025) (parametric) was fitted on the training set to remove batch effects. Subsequently, Z-score normalization parameters (mean and standard deviation) derived from the training set were calculated and applied to the test set.

A nested cross-validation framework was implemented for robust feature selection and hyperparameter tuning, consisting of 5 repeats in the outer loop and 10 folds in the inner loop. Within this framework, six distinct feature selection algorithms were executed: Random Forest (via randomForest package v4.7-1.1), XGBoost (v1.7.5.1), Gradient Boosting Machine (GBM; via gbm package v2.1.8.1), LASSO (via glmnet package v4.1-7), Boruta (v8.0.0), and Decision Tree (via rpart package v4.1.19). Consensus features were rigorously defined as those selected by at least four of the six algorithms. Furthermore, these consensus features were required to appear in over 50% of the internal cross-validation folds and to achieve an empirical *p*-value of less than 0.05 following Benjamini–Hochberg corrected permutation testing (1000 permutations).

To mitigate class imbalance, the class weights in the Random Forest algorithm were set to the inverse class frequency, while the scale_pos_weight parameter in XGBoost was adjusted based on the positive-to-negative sample ratio. The final model was trained on the entire training set using the selected consensus features and the optimized hyperparameters, and its performance was ultimately evaluated on the held-out independent test set.

The six algorithms yielded the following number of candidate genes: Random Forest (RF) initially selected 20 important genes; LASSO regression refined the set to 30 feature genes; XGBoost and Gradient Boosting Machine (GBM) identified 16 and 26 significant genes, respectively; the Boruta algorithm rigorously confirmed 12 core genes; and the Decision Tree (DT) model revealed the 10 most critical genes in the classification path.

### 2.9. Functional Enrichment, Network, and Downstream Analysis

Gene Ontology (GO) Biological Process and KEGG pathway enrichment analyses were performed using the clusterProfiler package (v4.8.0; https://yulab-smu.top/contribution-knowledge-mining/, accessed on 12 January 2025). Gene symbols were converted to ENTREZ IDs utilizing the org.Hs.eg.db database (v3.17.0; https://bioconductor.org/packages/org.Hs.eg.db, accessed on 23 12 January 2025), which successfully mapped 34 of the 36 differentially expressed genes (94.4%); the unmapped genes USP9Y and TTTY15 were excluded from the analysis. The background gene set was defined as all detected genes (18,542 genes with raw counts > 0 in at least one cell). The enrichment parameters were set as follows: pAdjustMethod = ‘BH’, pvalueCutoff = 0.05, qvalueCutoff = 0.05, minGSSize = 10, maxGSSize = 500, ont = ‘BP’. KEGG pathway analysis was executed with the enrichKEGG function (species = ‘hsa’) based on KEGG Release 106.0 (December 2023).

Pathway activity scores were calculated using Gene Set Variation Analysis (GSVA, v1.48.0; https://bioconductor.org/packages/GSVA, accessed on 12 January 2025) on log-normalized expression matrices. After verifying the approximate normality of the GSVA scores, a Gaussian kernel (kc.df = ‘Gaussian’) was employed. The GSVA was run with parameters: method = ‘gsva’, mx.diff = TRUE, min.sz = 5, max.sz = 500. Differential pathway activity between groups was then tested using the limma package (v3.56.0; https://bioconductor.org/packages/limma, accessed on 12 January 2025). The design matrix incorporated both group and donor effects, and intra-sample correlation was controlled for using the duplicateCorrelation function.

### 2.10. Statistical Analysis and Reproducibility

All analyses were performed in R (version 4.5.1). Key package versions are provided in Supplementary Table S3. To ensure the complete reproducibility of our analytical workflow, our approach implemented a systematic framework with the following key components:

Computational Reproducibility: Fixed random seeds were systematically applied to all stochastic processes throughout the analysis pipeline, including data partitioning (seed = 123), permutation tests (seed = 456), and bootstrap resampling (seed = 789). This ensures that all probabilistic computations yield identical results across independent executions.

Statistical Rigor: Statistical methods were selected based on rigorous diagnostic checks. Normality of data distributions was assessed using Shapiro–Wilk tests, guiding the choice between parametric and non-parametric approaches. For all hypothesis testing, a consistent multiple comparison correction strategy was implemented using the Benjamini–Hochberg false discovery rate (FDR) procedure, with a uniform significance threshold of FDR < 0.05. Effect sizes accompanied by 95% confidence intervals are reported for primary outcomes to provide quantitative measures of biological relevance beyond statistical significance.

Analytical Transparency: All critical analytical decisions, including quality control thresholds, preprocessing parameters, and algorithm configurations, are explicitly documented in their respective methodological sections. This comprehensive documentation enables the exact reconstruction of our analytical pathway from raw data to final results.

Verification Framework: Key analytical steps were validated through independent methodological approaches, including cross-validation in machine learning, bootstrap resampling for stability assessment, and comparison of multiple computational tools where applicable.

### 2.11. Validation and Sensitivity Analyses

To ensure the robustness of our findings, several validation and sensitivity analyses were performed.

Mitochondrial Gene Content Threshold. A sensitivity analysis was conducted to evaluate the impact of mitochondrial gene content thresholds (ranging from 5% to 30%) on cell retention and the identification of the HOX subpopulation. The proportional representation of HOX cells remained stable (21–27%) across all thresholds, confirming that the 20% cutoff used in our primary analysis effectively removed low-quality cells without excluding biologically relevant populations (Supplementary Figure S1).

Batch Correction Metrics. The performance of the Harmony batch correction algorithm was assessed using multiple complementary metrics. These included the reduction in batch-associated variance along PC1, the decrease in the number of batch-specific differentially expressed genes (DEGs), and improvements in the average Silhouette Width and kBET acceptance rate. The results demonstrate that Harmony effectively removed technical variability while preserving biological signal (Supplementary Figure S2).

Differential Expression Concordance. The robustness of DEG identification was evaluated by comparing results from three independent methods: Seurat (Wilcoxon test), MAST (mixed model), and pseudobulk analysis with edgeR. A high degree of concordance was observed, with 86% of the significant genes identified by Seurat also confirmed by both MAST and edgeR. The top 20 consensus DEGs are listed in Supplementary Table S4.

## 3. Results

### 3.1. Cellular Heterogeneity of Oxidative Stress Following Acute Myocardial Infarction at Single-Cell Resolution

**Figure 1:**
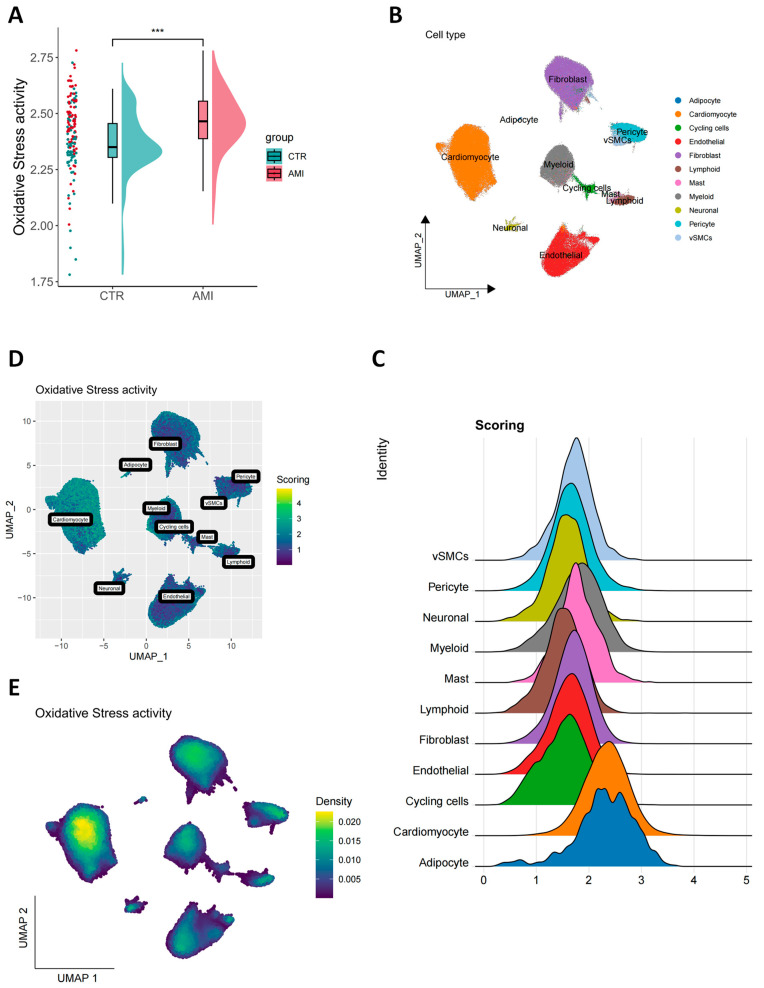
Oxidative stress activity in cardiomyocytes post-AMI. (**A**) Comparison of oxidative stress activity between the CT group and the AMI group. Based on bulk RNA-seq data analysis, oxidative stress activity in the AMI group is significantly higher than in the CT group (*** *p* < 0.001). (**B**) Annotation of AMI samples into 11 unique cell types using UMAP. (**C**) The total score of oxidative stress activity in 10 cell types. (**D**,**E**) The UMAP plot shows the distribution of oxidative stress activity in all cells of the AMI samples.

To systematically validate bulk sequencing findings and establish a phenotypic foundation for single-cell resolution analysis, a comparative analysis of bulk RNA sequencing data was first conducted. Myocardial tissue from the acute myocardial infarction (AMI) group exhibited significantly elevated levels of oxidative stress activation compared to the control group (*p* < 0.001, Figure 1A), thereby providing a phenotypic basis for subsequent single-cell investigation. This study integrated five independent single-cell transcriptomic datasets from the GEO database. Following systematic quality control, 190,566 high-quality cells were retained for subsequent analysis, with 1175 cells (approximately 0.61%) excluded. For disease-specific profiling, all control cells were removed, resulting in a final dataset of 149,090 cells from AMI samples. The distribution of these high-quality cells across conditions and donors is summarized in Table 1, providing transparency for donor-level analyses. A summary of post-quality control cell counts by donor and condition is provided in the main text (see Supplementary Table S2), enhancing transparency for donor-level modeling. Batch effects were effectively mitigated through Harmony normalization combined with Seurat-based clustering, leading to a significant reduction in technical variation post-integration (Figure S1A). This process identified 16 transcriptomically distinct cell clusters, which were annotated into 11 major cell types (Figure 1B and Figure S1B). Each cell type exhibited distinct signature gene expression profiles (Figure S1C). At the single-cell level, a preliminary comparison suggested the activation of oxidative stress was further corroborated: oxidative stress-related genes were significantly upregulated in AMI tissues compared to normal samples (*p* < 0.001, Figure S1D), which was consistent with the bulk transcriptomic results. As the primary evidence for subsequent analyses, a donor-level pseudo-bulk differential expression analysis was performed. To quantify the contribution of different cell types to overall oxidative stress, a composite oxidative stress score was calculated using five independent single-cell scoring methods (AUCell, UCell, singscore, ssGSEA, and Seurat’s AddModuleScore). Scores from each method were first Z-score normalized to ensure comparability, and a weighted average was computed based on the inverse variance of each method, thereby assigning higher weights to more stable scoring approaches. The final weight distribution across gene set sources was as follows: REACTOME (34.95%), WP (24.27%), HALLMARK (19.54%), KEGG (12.52%), and GOBP (8.72%). The robustness of this synthesis strategy was evaluated by comparing four alternative composite approaches (Simple_Avg, ZScore_Avg, Weighted_Avg, Rank_Avg), all of which showed high concordance (Pearson r = 0.8898–0.9772). Correlation analysis between individual gene set scores and the final composite score further supported the integration strategy (HALLMARK r = 0.4398, KEGG r = 0.3525, REACTOME r = 0.5905, GOBP r = 0.2948, WP r = 0.4930; all *p* < 0.001). Cell-type-specific comparisons based on the composite score identified adipocytes and cardiomyocytes as major oxidative stress “hotspots,” with scores significantly exceeding those of other cell types. Myeloid cells and mast cells also displayed notable stress activity (Figure 1C and Figure S1E). UMAP visualization revealed a gradient distribution of oxidative stress scores across cell types, forming spatially defined high-score regions that strongly overlapped with cardiomyocyte localization (Figure 1D,E), indicating a central role for cardiomyocytes in the AMI-associated oxidative stress response. Further functional analysis confirmed significant transcriptional reprogramming in post-AMI cardiomyocytes (between-group comparison *p* < 0.0001, Figure S1F). Based on these findings, a refined subpopulation analysis was conducted to resolve the intrinsic heterogeneity of cardiomyocytes.

### 3.2. Heterogeneity of Oxidative Stress in Cardiomyocytes and Identification of HOX Subpopulations

**Figure 2:**
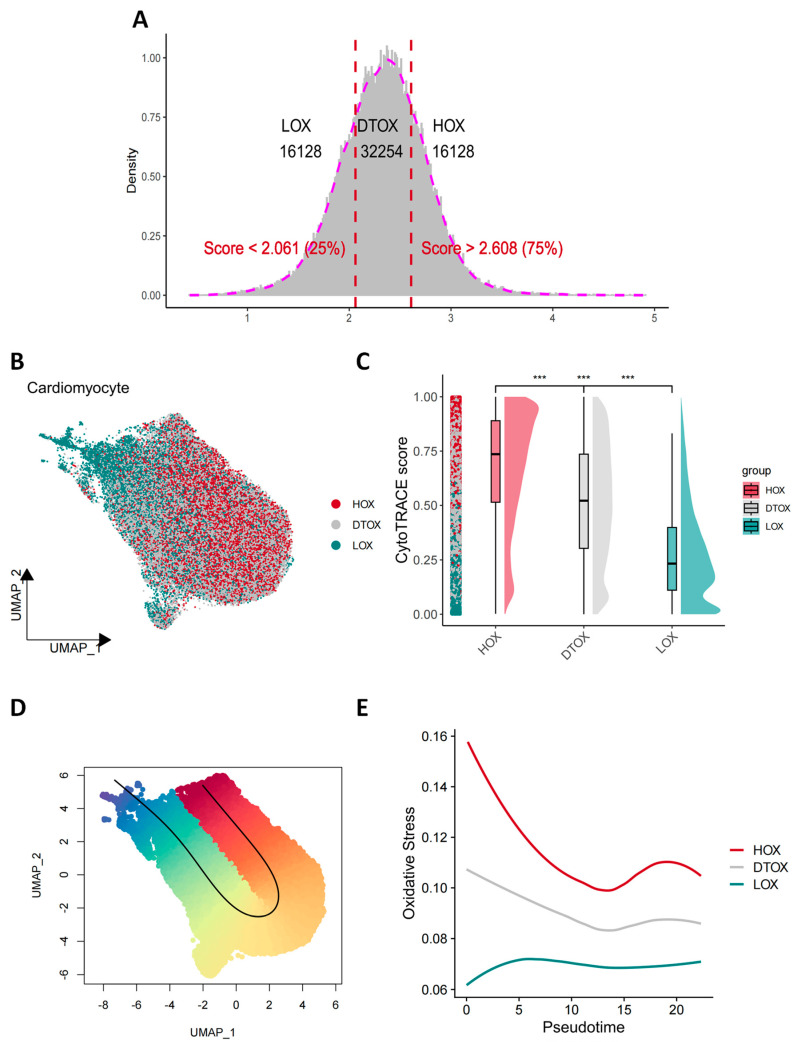
Heterogeneity of cardiomyocytes. (**A**) Oxidative stress activity histogram showing the distribution of oxidative stress scores across different conditions (HOX, DTOX, LOX). The dashed vertical lines indicate the score thresholds (Score < 2.061 and Score > 2.608). (**B**) UMAP plot of cardiomyocytes from different groups (HOX, DTOX, LOX). (**C**) Boxplot comparing CytoTRACE scores across different groups (HOX, DTOX, LOX) (*** *p* < 0.001). (**D**) UMAP plot showing the trajectory of cells based on their pseudotime, with the color gradient representing the progression of oxidative stress activity. (**E**) Line plot showing the change in oxidative stress activity over pseudotime, with different colors representing HOX, DTOX, and LOX groups. The plot illustrates the trend of oxidative stress activity as cells progress through pseudotime.

The robustness of HOX subpopulation identification was further supported by the consistent detection of its marker genes across multiple differential expression methods (Supplementary Table S4). The analysis commenced with an examination of the weighted composite oxidative stress scores across 64,510 cardiomyocytes. Based on quartile-derived thresholds, the cells were stratified into three distinct groups: a Low Oxidative Stress (LOX) group (score < Q1 = 2.061), a Dynamic Transient Oxidative Stress (DTOX) group (Q1 ≤ score ≤ Q3), and a High Oxidative Stress (HOX) group (score > Q3 = 2.608). This stratification resulted in 16,128 LOX, 32,254 DTOX, and 16,128 HOX cells, with their distribution visualized in a histogram defining the groups (Figure 2A). UMAP visualization revealed a sequential spatial distribution of these groups, where DTOX cells occupied an intermediate position between LOX and HOX clusters. This spatial pattern supported the hypothesis of a sustained dynamic continuum of oxidative stress (Figure 2B and Figure S2A). Cellular immaturity and plasticity were subsequently assessed using CytoTRACE. A notable spatial colocalization was observed between high CytoTRACE scores and the HOX subpopulation on the UMAP (Figure S2B,C). Consistently, CytoTRACE scores were found to be significantly higher in HOX cells compared to both DTOX and LOX cells (*p* < 0.001, Figure 2C). While both CytoTRACE and trajectory analysis indicated that HOX cells exhibit higher plasticity, it is important to note that these data provide only correlational evidence and do not establish causality for dedifferentiation or reprogramming. Future functional experiments, such as genetic interventions and lineage tracing, will be necessary to validate the mechanistic link between the HOX state and cellular plasticity. Multivariate correlation analysis further revealed a moderate positive correlation between oxidative stress scores and CytoTRACE scores (Pearson r = 0.52, *p* < 0.001; Figure S2D,E), with the majority of HOX cells residing in the high-stress, high-plasticity quadrant. Pseudotime trajectory analysis illustrated an ordered progression from LOX through DTOX to HOX, with HOX cells predominantly enriched at the trajectory endpoint (Figure 2D). Along this trajectory, oxidative stress activity demonstrated a biphasic dynamic, initially decreasing before rising sharply to peak at the HOX-enriched end (Figure 2E). This pattern indicates that the myocardial stress response to AMI follows a phased, dynamic process.

### 3.3. HOX as a Cellular Communication Hub with Metabolic Reprogramming Pheno-Types

**Figure 3:**
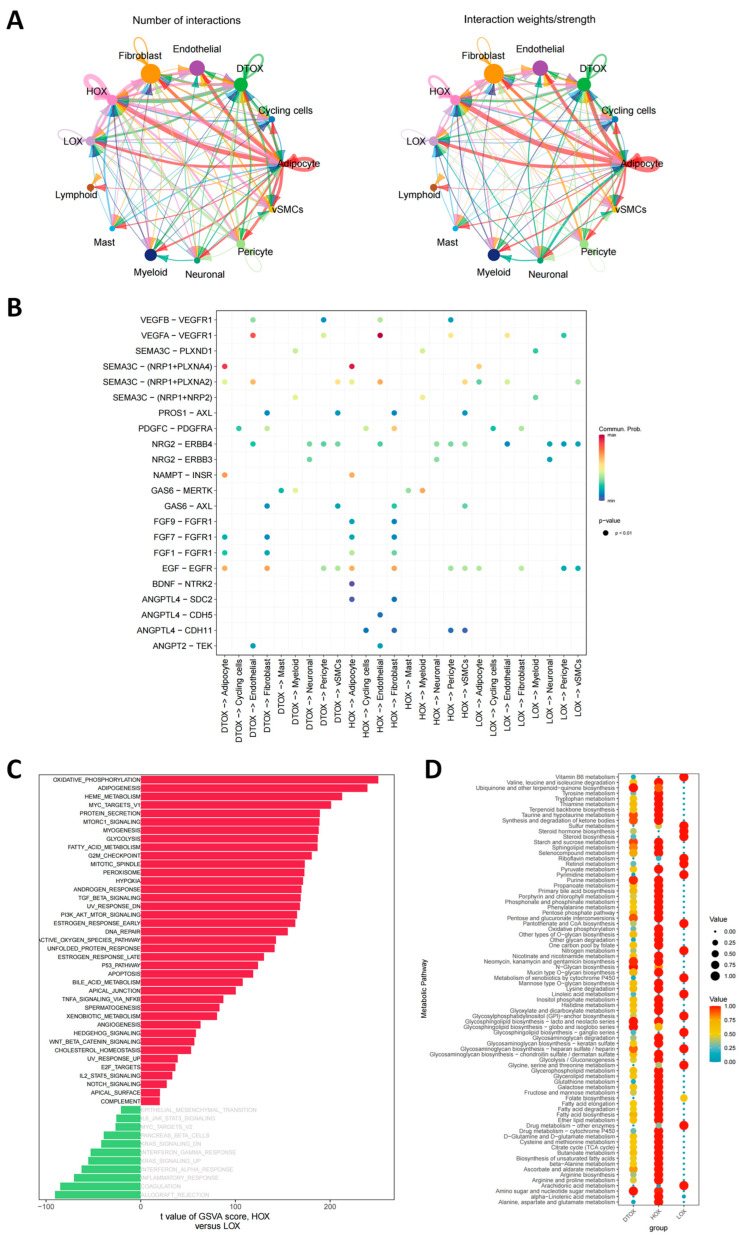
Communication and metabolism in HOX population. (**A**) Interaction network diagram illustrating the number and intensity of cellular communications among HOX, LOX, DTOX, and other cell types within AMI tissues. Each node represents a cell type, distinguished by a unique color: HOX (pink), LOX (light purple), DTOX (green), Fibroblast (orange), Endothelial (purple), Cycling cells (cornflower blue), Myeloid (navy blue), Lymphoid (brown), Pericyte (light green), Adipocyte (red), Mast (sky blue), vSMCs (yellow) and Neuronal (forest green). (**B**) Ligand-receptor interactions between HOX, LOX, DTOX cells, and various other cell types. (**C**) Enrichment of HOX cells in various metabolic pathways. The pathways are ranked based on their significance, with positive values indicating enrichment in HOX and negative values in LOX. (**D**) Significant changes in AMI-related metabolic pathways involving HOX cells. The bubbles’ size represents the pathways’ value, and the color gradient indicates the activity level for each metabolic pathway in different groups.

To evaluate the role of HOX cardiomyocytes within the myocardial infarction microenvironment, an intercellular communication network was constructed. Holistic network analysis revealed that HOX cardiomyocytes exhibited topological hub properties, establishing extensive interactions with fibroblasts, endothelial cells, adipocytes, and myeloid cells (Figure 3A). Ligand-receptor profiling further indicated that HOX cells possessed strong signaling input and output capacity. These cells showed elevated expression of pro-angiogenic and survival ligands such as VEGF, FGF, EGF, NGF, and GAS6, as well as enriched receptors involved in metabolic and structural sensing, including Visfatin, IGF, BMP, and POSTN. Potential autocrine and paracrine feedback loops were also identified, such as the NRG-ErbB pathway (Figure S3A). Quantitative analysis demonstrated a significantly greater number of ligand-receptor interactions in HOX compared to LOX conditions (Figure S3B–D). HOX cells and adipocytes jointly emerged as core communication nodes within the infarcted microenvironment, suggesting their coordinated role in regulating cardiac stress and metabolic responses under pathological conditions (Figure S3B). Refined pathway analysis revealed that HOX cells transmitted functional signals through specific pathways, including VEGFA-VEGFR1, SEMA3C-(NRP1+PLXNA4), and EGF-EGFR, which are known to influence vascular permeability [23], adipocyte behavior [24], and fibroblast activation [25] (Figure 3B). Functional impact scoring analysis showed that under normal conditions, cellular functional indicators declined with increasing oxidative stress (LOX > DTOX > HOX). In contrast, under AMI conditions, HOX cells exhibited elevated functional indicators that surpassed those of the normal group, suggesting an adaptive or remodeling-related functional enhancement within the pathological microenvironment (Figure S3E). Pathway activity comparison between HOX and LOX cells using GSVA revealed a “metabolic activation–immune suppression” profile in the HOX group. Specifically, HOX cells upregulated pathways related to oxidative phosphorylation, lipid synthesis, heme metabolism, MYC target genes, and protein secretion, while downregulating pathways involved in acquired immunity, including transplant rejection, coagulation, and certain inflammatory responses (Figure 3C). The upregulation of MYC target genes implied that core transcriptional programs may coordinate both enhanced metabolism and immune suppression. Further systemic metabolic analysis confirmed synchronous activation of multiple energy and auxiliary metabolic routes in HOX cells, including the TCA cycle, glycolysis/gluconeogenesis, lipid biosynthesis, porphyrin metabolism, and glutathione metabolism (Figure 3D). Together, these findings indicate that HOX cardiomyocytes undergo global metabolic reprogramming within the infarcted heart.

### 3.4. Analysis of HOX Transcriptional Regulatory Networks Based on hdWGCNA

**Figure 4:**
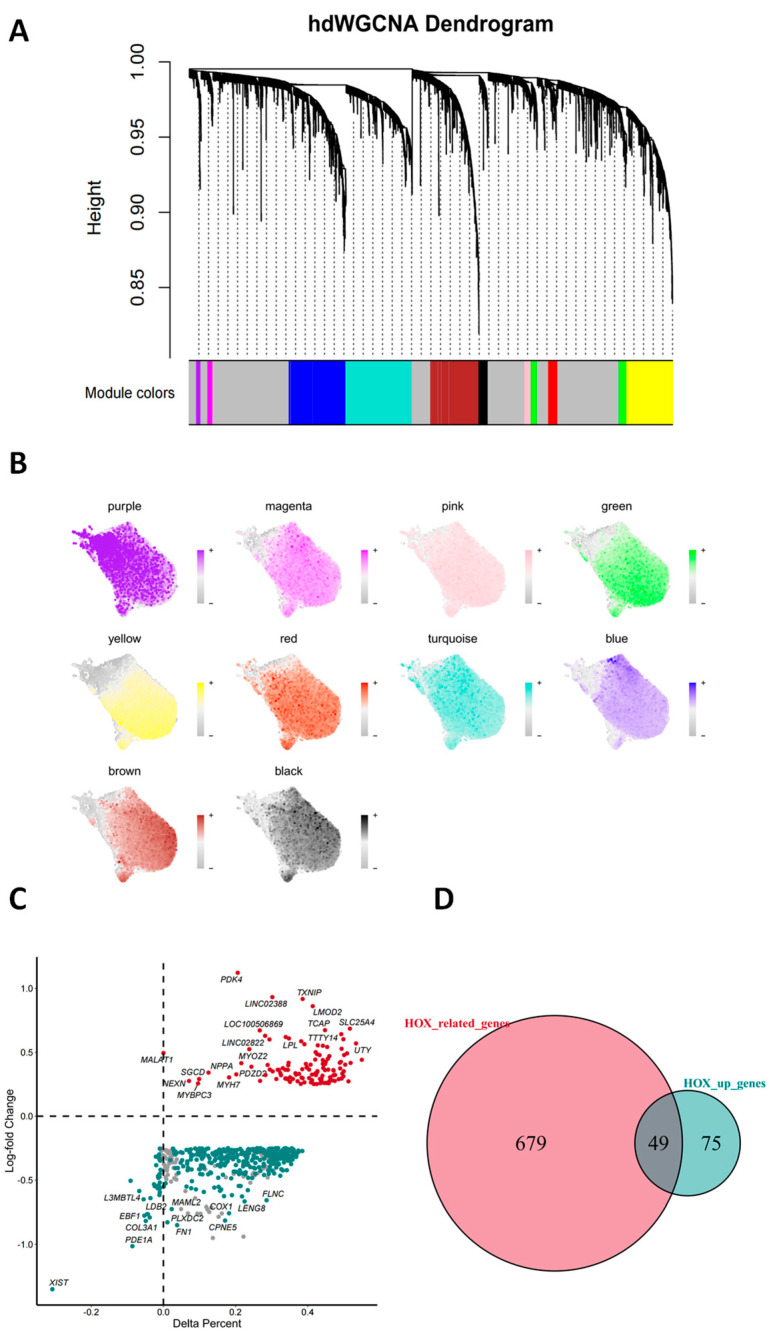
Deciphering HOX cell functions through hdWGCNA and oxidative stress gene analysis. (**A**) Hierarchical clustering dendrogram of gene modules identified by hierarchical, weighted gene co-expression network analysis (hdWGCNA). The dendrogram shows how genes are grouped into different modules based on co-expression patterns. (**B**) UMAP plot showing the expression of 11 gene modules across the samples. Each module is represented by a different color, reflecting the gene expression distribution in each module across the other samples. (**C**) Volcano plot showing the log2 fold change versus delta per cent of gene expression in HOX Vs. LOX groups. Significant genes with high fold change are highlighted in red, while genes with low fold change are highlighted in blue. (**D**) Venn diagram showing the overlap between genes related to HOX and those with significant upregulation in the HOX group.

A weighted gene co-expression network (WGCNA) was constructed to identify gene modules associated with oxidative stress states in cardiomyocytes. The soft threshold power β was set to 9 based on a scale-free topology fit index exceeding 0.8 (Figure S4A). The resulting network was partitioned into 10 distinct modules, among which the blue module exhibited a strong positive correlation with the HOX phenotype, while the blue-green module correlated with the LOX group (Figure 4A,B). Genes within the blue module demonstrated both higher average expression and broader expression coverage in HOX cells compared to other subpopulations (Figure S4B). Differential expression analysis between HOX and LOX cells revealed significant gene expression remodeling in the HOX group (Figure 4C), with upregulation of multiple metabolism and stress-related genes such as PDK4, TXNIP, EIF1B, SLC25A4, and CHCHD10. Cross-referencing the differentially expressed genes (DEGs) with WGCNA modules identified 49 dual-validated core HOX genes (Figure 4D). The pathological relevance of this core gene set was further assessed. Significant positive correlations were observed between oxidative stress scores and the expression of EIF1B, SLC25A4, TXNIP, CHCHD10, and PDK4. These genes were mapped into a functional regulatory network, where EIF1B is implicated in protein synthesis [26], SLC25A4 in energy metabolism and transport [27], TXNIP in oxidative stress sensing [28], CHCHD10 in mitochondrial function maintenance [29], and PDK4 in core metabolic reprogramming [30] (Figure S4C). Disease and pathway enrichment analysis indicated that the core gene set was significantly enriched in cardiomyopathy-related diseases (Figure S4D). Gene Ontology (GO) analysis highlighted biological processes including muscle system processes, cardiac tissue development, fatty acid β-oxidation, and glucose homeostasis. Cellular component analysis further emphasized mitochondria-associated structures and myofibrillar units (Figure S4E). These results suggest that mitochondria-mediated energy metabolism and myofibrillar structural integrity constitute the core biological basis of the HOX phenotype.

### 3.5. Screening Key Feature Genes for Acute Myocardial Infarction Using Multi-Algorithm Ensemble Models

**Figure 5:**
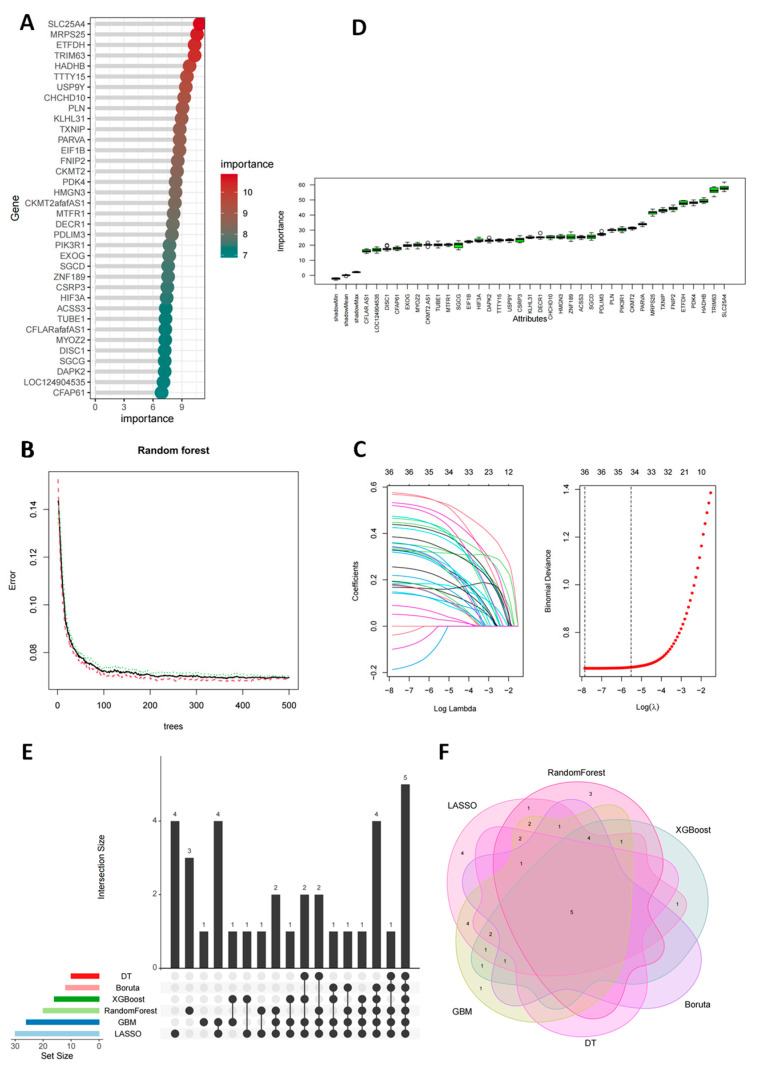
Identify optimal feature genes for AMI using multiple machine-learning algorithms (**A**) The top 20 feature genes with an importance score greater than 8.0, as identified by the Random Forest algorithm. (**B**) The Out-of-Bag (OOB) error rate of the Random Forest model. The lines represent the overall OOB error (black), the error rate for the non-AMI control group (con, green), and the error rate for the AMI group (treat, red), demonstrating model convergence and high performance. (**C**) LASSO coefficient paths for feature gene selection. Each colored line represents the coefficient trajectory of a candidate gene across different regularization parameters. The left vertical dotted line indicates lambda.min (λ = 0.0004, optimal value with minimum cross-validation error), and the right vertical dotted line indicates lambda.1se (λ = 0.0040, largest λ within one standard error of minimum error). Numbers above indicate the count of genes with non-zero coefficients at corresponding λ values. Thirty potential diagnostic biomarkers for acute myocardial infarction (AMI) were chosen from 36 candidate feature genes obtained from LASSO analysis at lambda.min. (**D**) Twelve feature genes were identified using Boruta analysis. (**E**,**F**) Intersection analysis of the feature genes identified by six machine learning algorithms. (**F**) Venn diagram and (**E**) UpSet plot showing the overlaps among genes selected by RandomForest (RF), LASSO, XGBoost, Boruta, Decision Tree (DT), and Gradient Boosting Machine (GBM). Different colors represent distinct algorithms, numbers indicate gene counts for each algorithm and their intersections, and bar heights show set sizes. The analysis revealed five optimal feature genes consistently identified across multiple algorithms.

To identify key diagnostic biomarkers for AMI with high confidence, this study employed an integrative machine-learning framework encompassing six distinct algorithms. This multi-step screening process yielded robust and convergent evidence for gene selection. The Random Forest algorithm identified 20 high-importance genes (Figure 5A), with model error converging rapidly (Figure 5B). Subsequent LASSO regression refined the candidate set to 30 feature genes with positive coefficients (Figure 5C). Validation using gradient-boosting models—XGBoost and GBM—highlighted 16 and 26 significant genes, respectively (Figure S5A,C). The Boruta algorithm provided rigorous confirmation of 12 core genes (Figure 5D), while a Decision Tree model pinpointed the 10 most critical genes for classification (Figure S5B).

Cross-analysis of all outputs from the six algorithms revealed a consistent core set of five genes: TRIM63, ETFDH, TXNIP, CKMT2, and PDK4 (Figure 5E,F). These consensus genes exhibited elevated expression in independent cohorts, demonstrating high stability and significance across diverse computational frameworks. Notably, TXNIP and ETFDH were also identified as core HOX genes in our prior hdWGCNA analysis, further underscoring their pivotal role in AMI pathology.

### 3.6. Validation of Key Gene Expression and Assessment of Diagnostic Value

**Figure 6:**
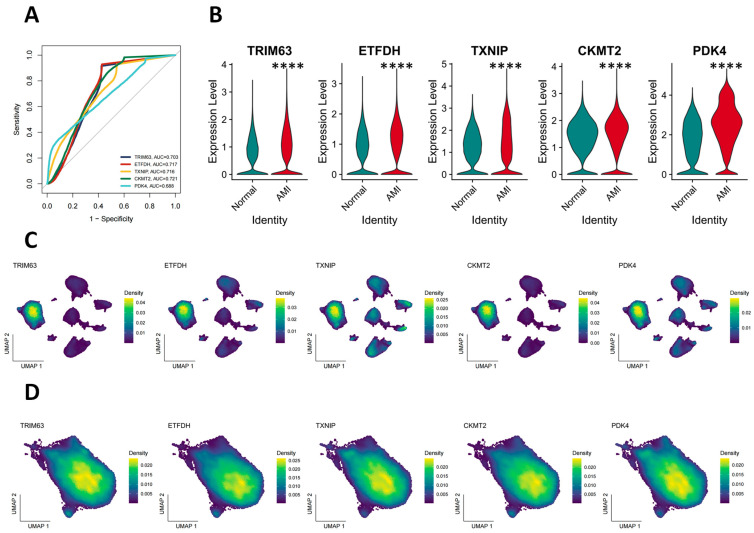
Assessing *TRIM63*, *ETFDH*, *TXNIP*, *CKMT2*, and *PDK4* as predictive markers for AMI. (**A**) ROC curve analysis for the diagnostic performance of the selected feature genes (*TRIM63*, *ETFDH*, *TXNIP*, *CKMT2*, and *PDK4*). (**B**) Violin plots showing the expression levels of the feature genes (*TRIM63*, *ETFDH*, *TXNIP*, *CKMT2*, and *PDK4*) in normal and AMI samples (**** *p* < 0.0001). (**C**) UMAP plots show the AMI samples’ feature gene expression distribution (*TRIM63*, *ETFDH*, *TXNIP*, *CKMT2*, and *PDK4*). The color density reflects the expression level of each gene across the UMAP dimensions. (**D**) UMAP plots show the feature gene expression distribution (*TRIM63*, *ETFDH*, *TXNIP*, *CKMT2*, and *PDK4*) in cardiomyocytes post-AMI. The color density indicates the expression level of each gene across the UMAP dimensions.

The clinical relevance of the five consensus genes was validated using three independent transcriptomics cohorts (GSE61145, GSE97320, GSE19339), comprising a total of 62 samples. Receiver operating characteristic (ROC) curve analysis demonstrated significant discriminatory power for all five genes (AUCs > 0.5, *p* < 0.001), with AUC values as follows: CKMT2 0.721, ETFDH 0.717, TXNIP 0.716, TRIM63 0.703, and PDK4 0.688 (Figure 6A). Expression analysis confirmed that all five genes were significantly upregulated in the acute myocardial infarction group compared to controls (*p* < 0.0001; Figure 6B). Both tissue-level and single-cell-level analyses indicated cardiomyocyte-specific expression of these genes (Figure S6A,B). Further single-cell resolution analysis revealed significant enrichment of all five genes in the HOX subpopulation (Figure S6C, Figure 6D, respectively), with strong spatial colocalization observed in UMAP visualizations (Figure 6C). These findings support their role as stable molecular markers of the HOX phenotype. Functionally, TRIM63 expression indicated activation of protein degradation and remodeling pathways, while ETFDH and PDK4 jointly suggested a metabolic shift toward increased fatty acid oxidation and suppressed glucose oxidation. TXNIP expression was consistent with heightened oxidative stress damage. Together, these molecular profiles confirm that HOX cells exhibit distinct metabolic and stress-related phenotypic characteristics. In summary, this study conducted a multi-level analysis of myocardial oxidative stress heterogeneity following acute myocardial infarction. It identified and validated a set of core genes and pathways strongly associated with the HOX phenotype. HOX cells were found to carry transcriptional signatures related to intercellular communication and metabolic reprogramming, suggesting a potential central role in microenvironmental information exchange and regulation. However, further functional validation is required to establish causal relationships underlying the “high oxidative stress–high plasticity” phenotype observed in HOX cells.

## 4. Discussion

### 4.1. The HOX Subpopulation: A Novel State of High Plasticity and Metabolic Stress

One key factor distinguishing HOX cells from other cardiomyocyte populations was their transcriptional signature. The systematic comparison with previously described cardiomyocyte subtypes (metabolic, stressed, failing, and proliferative) revealed limited overlap (Jaccard similarity < 0.364), with PDK4 being the primary shared marker. However, HOX cells exhibited 2.3-fold higher PDK4 expression than classical metabolic cardiomyocytes, supporting their classification as a novel subpopulation defined by a unique oxidative stress metabolic phenotype. The extent to which these signatures influenced cell fate or function remained to be validated by functional experiments. Importantly, donor variability analysis showed low inter-donor differences (ICC = 0.043), indicating the robustness of HOX identification across individuals.

### 4.2. Metabolic-Immune Dichotomy and Intercellular Communication

GSVA analysis revealed the phenomenon of “coexisting metabolic activation and downregulated immune signaling”. This phenomenon represented a core characteristic of the HOX phenotype. The concurrent upregulation of oxidative phosphorylation and glycolysis, while seemingly contradictory, may reflect a state of ‘metabolic stress’ where cells attempt to utilize all available energy sources under pathological conditions. The synergistic upregulation of oxidative phosphorylation, fatty acid metabolism, and MYC target gene pathways provided the essential energy and biosynthetic foundation. This foundation allowed cells to maintain a high-energy-consumption plasticity state under high-stress conditions. The transcriptional downregulation of acquired immune-related pathways suggested potential immune regulatory or suppressive mechanisms.

This phenomenon may reflect changes in immune cell proportions or differences in local inflammatory states. These possibilities require further validation. Researchers should validate them with immune cell function assays and antigen presentation experiments. The “metabolic activation-immune suppression” feature combination in HOX cells shares similarities with adaptive strategies used by certain tumor cells [31]. Those tumor cells adopted similar strategies when they faced stress. Cells in chronic inflammation or during tissue repair may show similar metabolic reprogramming [32]. These cells meet high energy demands and modulate the local immune microenvironment [33]. Researchers not only need to determine whether this phenotypic similarity reflects conserved regulatory logic across cell types, but also need to determine whether it represents an independently evolved adaptive response by cardiomyocytes under pathological stress. Validation requires comparative genomics and functional experiments. MYC acts as a key transcription factor in cell proliferation, metabolism, and immune regulation [34]. The upregulation of MYC target genes suggests that core transcriptional programs coordinate enhanced metabolism and immune suppression. These programs promote expression of genes in energy metabolism pathways and regulate inflammation-related signaling pathways [35]. Together, they drive the complex adaptive phenotype. This metabolic-immune signature may provide survival advantages to HOX cells in inflammatory environments. Researchers must confirm the ultimate fate of these cells with lineage tracing experiments. Future research focuses on validating whether cardiomyocytes use a tumor-like adaptive mechanism and on testing its functional significance. Based on current evidence, we propose that the HOX state reflects a “trade-off” strategy. This strategy reallocates resources between energy survival demands and immune homeostasis. In myocardial injury, the trade-off may cause HOX cells to temporarily suppress specific immune responses [36]. This suppression may reduce excessive inflammatory damage or promote tissue remodeling and may also prioritize energy for repair or self-survival. The concurrent upregulation of metabolic pathways and downregulation of immune pathways in our model supports this view. Subsequent studies must test this working hypothesis.

### 4.3. A Robust Multi-Gene Signature for AMI Diagnosis

Another key strength of this study lies in its use of a multi-level, multi-method computational biology strategy. The strategy established a convergent evidence chain from systems biology to precision biomarkers. The blue module identified through hdWGCNA analysis showed a strong correlation with the HOX phenotype. The module’s gene set showed significant overlap with the up-regulated gene set from differential expression analysis. Our study integrated two complementary dimensions: co-expression regulatory networks and differential expression. This integration yielded a core HOX signature of 49 genes. The approach combined co-expression network topology with differential expression analysis. It enabled more comprehensive and robust identification of key gene sets driving cell subpopulation phenotypes. The approach offered greater credibility and specificity than single methods. Enrichment analysis showed significant association of this gene set with cardiomyopathy. GO enrichment analysis showed that these 49 genes participated in energy metabolism and myofibrillar structure maintenance. GO analysis also showed that the genes could be subdivided into submodules for specific cellular signaling, stress responses, or structural remodeling. These results suggested complex functional regulatory mechanisms in HOX cells. Cross-validation was performed using six machine learning algorithms with distinct principles, including Random Forest, LASSO, and XGBoost. Five genes were consistently selected by all six algorithms. The remaining 44 core HOX genes did not meet this criterion. Further analysis showed that the five consistent genes had superior expression stability and pathway enrichment characteristics. The other 44 genes were more often involved in biologically heterogeneous processes such as cellular signaling and structural remodeling. This involvement likely explained their inconsistent performance in algorithmic screening. These five genes exhibit highly synergistic effects. *PDK4* inhibits glucose oxidation and promotes the conversion of metabolic substrates into fatty acids. *ETFDH* acts as a key enzyme in fatty acid β-oxidation [37]. *CKMT2* maintains cellular energy homeostasis by synthesizing phosphocreatine from ATP [38]. *TXNIP* mediates oxidative stress responses [39]. *TRIM63* participates in protein quality control [40]. Notably, *PDK4* and *TXNIP* have extensive evidence linking them to myocardial metabolic reprogramming and oxidative stress [41]. This evidence comes from models such as ischemia–reperfusion injury and heart failure [42]. This study identifies their co-expression and synergistic effects within single-cell HOX subpopulations. This finding further underscores their pivotal role in acute myocardial infarction pathology. Correlation analysis revealed that these HOX signature genes show low correlation with traditional clinical biomarkers like troponin (e.g., PDK4: r = 0.182), suggesting they capture complementary biological information related to cellular metabolic stress rather than cardiomyocyte necrosis. Within the sample and analytical framework of this research, these genes collectively indicate a metabolic-oxidative stress-related pathological tendency. These genes constitute a pathophysiological pathway that warrants further experimental validation. Their shared upstream regulatory factors also merit in-depth investigation.

The five key genes identified in this study had a robust computational biology foundation. The genes also showed clear translational potential. ROC analysis showed strong diagnostic discriminatory power for acute myocardial infarction (AMI). An independent sample validated that the genes were specifically upregulated in AMI tissue. Single-cell validation precisely localized these markers to the most pathologically representative HOX cell subpopulations. This localization increased their biological plausibility as biomarkers. This integrated five-gene “molecular signature panel” provides more comprehensive disease state information than traditional single biomarkers. The panel captures multiple interrelated aspects of the core pathophysiology of acute myocardial infarction. The approach holds promise for improving diagnostic sensitivity and specificity. The panel may enable early identification of specific patient cohorts. These cohorts include patients whose infarct-related arteries have spontaneously reperfused but who retain myocardial edema and oxidative stress. Conventional cardiac enzyme biomarkers often overlook this group. This oversight creates a critical blind spot in routine testing. Researchers should investigate whether the proportion of HOX cell subpopulations or the expression levels of their characteristic genes correlate with long-term outcomes. These outcomes include incidence of heart failure, severity of left ventricular remodeling, and risk of malignant arrhythmias [43]. Such correlations may provide novel cell biological evidence for risk stratification in AMI. At the therapeutic level, HOX subpopulations represent a promising repository of precision treatment targets. Our findings suggest that future interventions should not only target oxidative stress. They should also take cellular heterogeneity into account. Designing metabolic interventions that target unique metabolic dependencies, such as fatty acid oxidation, offers an innovative path. Potential strategies include *PDK4* inhibitors, *ETFDH* activators, and *TXNIP* antagonists. Precise targeting of HOX cells may require developing cell-specific delivery systems. Such systems aim to maximize therapeutic efficacy and minimize off-target effects [44].

### 4.4. Study Limitations and Future Directions

This study has several limitations. Most conclusions are based on computational inferences from transcriptomic data. The mechanisms by which key genes shape the HOX phenotype require functional validation. Such validation requires cardiomyocyte-specific transgenic animal models. Examples include conditional gene knockout or overexpression using the Cre-LoxP system. Single-cell sequencing has limited efficiency in capturing rare cell subpopulations. The absence of dynamic data across multiple time points limits our ability to track HOX cell emergence. The absence of such data also limits understanding of HOX cell evolution. Lack of spatial transcriptomics data limits confirmation of the precise distribution of HOX cells within the infarct region. The lack also limits understanding of microenvironment interaction patterns and cell–cell communication. The diagnostic efficacy of the five signature genes requires further validation in prospective, multicenter cohort studies. These studies should use peripheral blood samples to assess clinical translational potential. Detecting myocardial-specific markers in peripheral blood faces dilution effects and sensitivity challenges. Researchers, therefore, need highly sensitive assay methods.

## 5. Conclusions

This study systematically analyzes oxidative stress heterogeneity in cardiomyocytes after acute myocardial infarction at single-cell resolution. The study identifies a unique HOX subpopulation that shows both high oxidative stress and high cellular plasticity. Transcriptomic analysis shows strong activation of oxidative phosphorylation and fatty acid metabolism in this subpopulation. These patterns indicate distinct metabolic reprogramming. We integrate six machine learning algorithms with cross-validation to identify and validate a core candidate gene set: *TRIM63*, *ETFDH*, *TXNIP*, *CKMT2*, and *PDK4*. The findings show that the HOX subpopulation plays a critical role in AMI pathophysiology through a “metabolic activation–immune suppression” program. This work provides a theoretical foundation for clarifying the molecular mechanisms of metabolic heterogeneity in AMI cardiomyocytes. It also supports the development of diagnostic biomarkers and the design of precision treatment strategies. Future studies should focus on the functional validation of these targets, employing spatial transcriptomics to resolve the spatiotemporal dynamics of HOX cells, and advancing the translational application of the diagnostic signature in large-scale prospective clinical cohorts.

## Figures and Tables

**Table 1:** Summary of post-quality control single-cell data used in this study.

Condition	Number of Donors	Number of Samples	Total Final Cells (Post-QC)
Normal	4	4	32,476
AMI	16	25	116,614
Overall	20	29	149,090

## Data Availability

The original datasets presented in this study are openly available in the NCBI GEO repository under the accession numbers GSE48060, GSE66360, GSE61145, GSE97320, and GSE19339.

## Supplementary Materials

The following supporting information can be downloaded at https://www.mdpi.com/article/10.3390/antiox14121435/s1. Table S1: dataset information; Table S2: clinical information; Table S3: software information; Table S4: gene comparison; Figure S1. Data Integration and Quality Assessment. (Related to main Figure 1); Figure S2. Supplementary Analysis of HOX Subpopulation Plasticity and Pseudotime Trajectory. (Related to main Figure 2); Figure S3. Detailed Dissection of Intercellular Communication by HOX Cells. (Related to main Figure 3); Figure S4. Functional Enrichment Analysis of the HOX Core Gene Module. (Related to main Figure 4); Figure S5. Feature Selection Results from Additional Machine Learning Algorithms. (Related to main Figure 5); Figure S6. Quantitative Expression of Core Feature Genes across Cardiomyocyte Subpopulations. (Related to main Figure 6).
