# Supplementary material for: Single-Cell Transcriptomics of Human Acute Myocardial Infarction Reveals Oxidative Stress-Associated Cardiomyocyte Subpopulations and Candidate Predictive Signatures
Source: Antioxidants (Basel). 2025 Nov 28;14(12):1435. doi: 10.3390/antiox14121435 (PMC12729884; doi:10.3390/antiox14121435)
Supplement: Supplementary file 1 [file antioxidants-14-01435-s001.zip › Supplementary Figures.pdf]

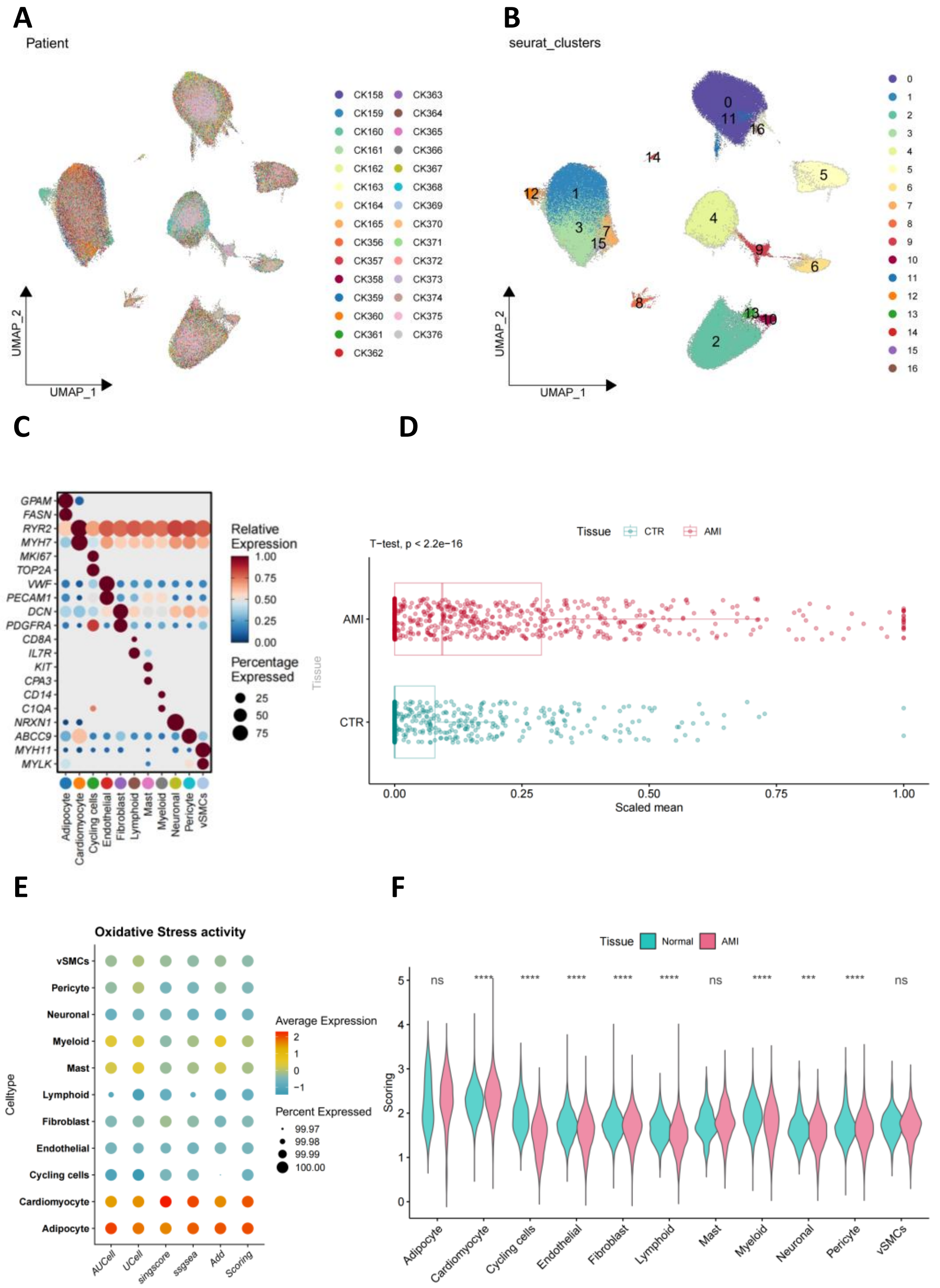

Supplementary Figure S1 Data Integration and Quality Assessment. (A) The cell distribution of the samples showed no significant batch effect. (B) Cell populations are categorised into 16 clusters. (C) Expression patterns of cell typing marker genes. (D) Comparison of scaled mean expression between Normal and AMI tissues. Single-cell sequencing analysis reveals significantly higher expression in the AMI group compared to the Normal group (***P < 0.001). (E) The average expression of oxidative stress activity across 10 cell types was determined using five methods. (F) Differential expression patterns of 11 cell types in normal and AMI.

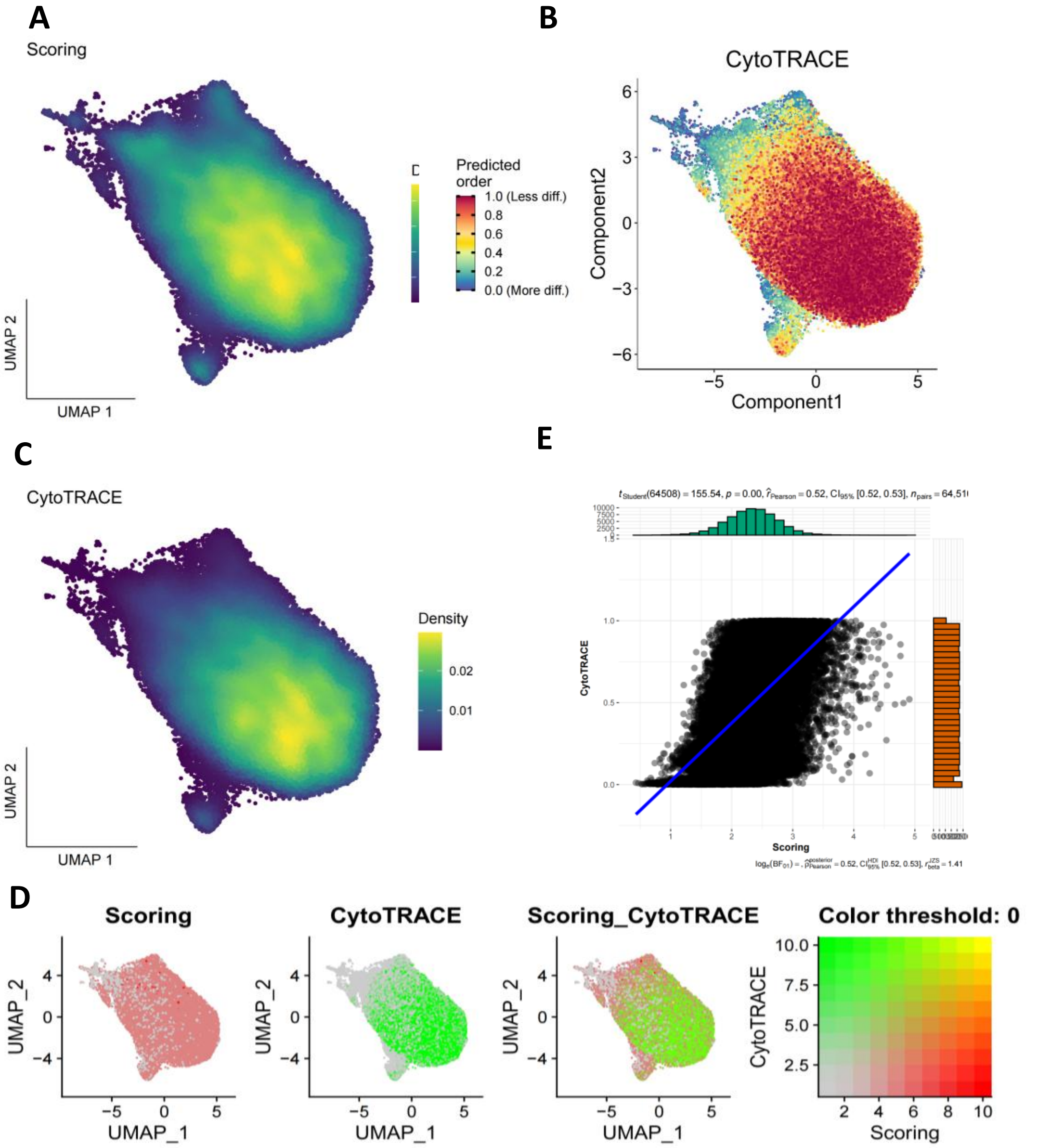

Supplementary Figure S2 Supplementary Analysis of HOX Subpopulation Plasticity and Pseudotime Trajectory. (A) UMAP plot coloured by oxidative stress scoring, showing the density distribution of oxidative stress activity across different groups. (B) UMAP plot of CytoTRACE scores, representing the predicted developmental ordering of cells based on CytoTRACE analysis. (C) The UMAP plot shows CytoTRACE values' density, highlighting the overall distribution of predicted cell states. (D) UMAP plots show the distribution of cells coloured by (from left to right) oxidative stress scoring, CytoTRACE scores, and the combined scoring of oxidative stress and CytoTRACE. (E) A scatter plot shows the correlation between oxidative stress scoring and CytoTRACE, with a regression line indicating the relationship between the two metrics.

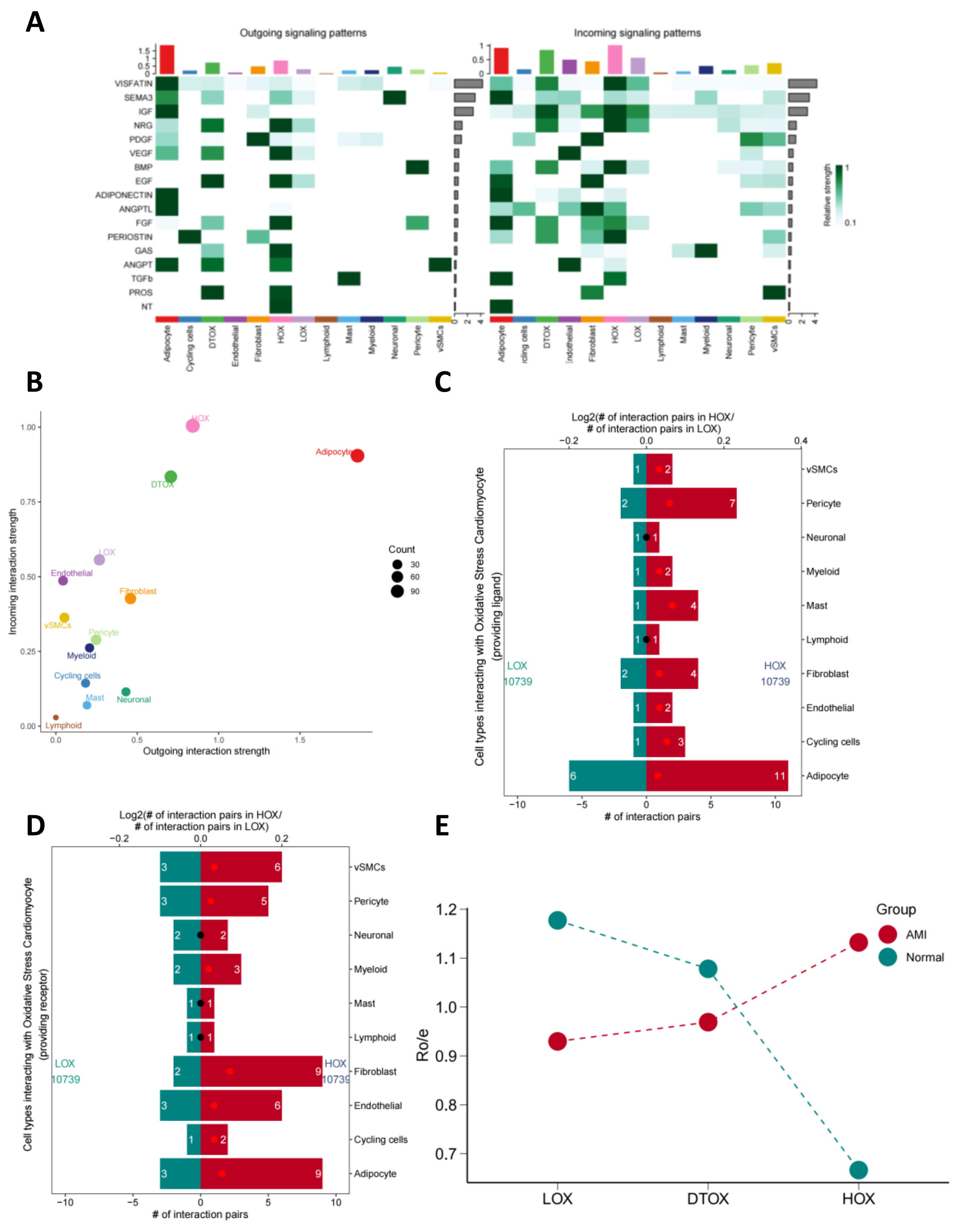

Supplementary Figure S3 Detailed Dissection of Intercellular Communication by HOX Cells. (A) Analysis of ligand-receptor interactions among different cell types. The heatmap shows the presence and intensity of signalling pathways between various cell types, with the colours representing different signalling intensities. (B) Evaluation of incoming and outgoing interaction intensities for different cell types. The size of the dots represents the count of interactions, with colour representing the interaction strength. (C) and (D) Analysis of ligand-receptor interactions between HOX, LOX, and other cell types using CellPhoneDB and snRNA-seq data. (E) Ro/e analysis of tissue enrichment for different cell populations.

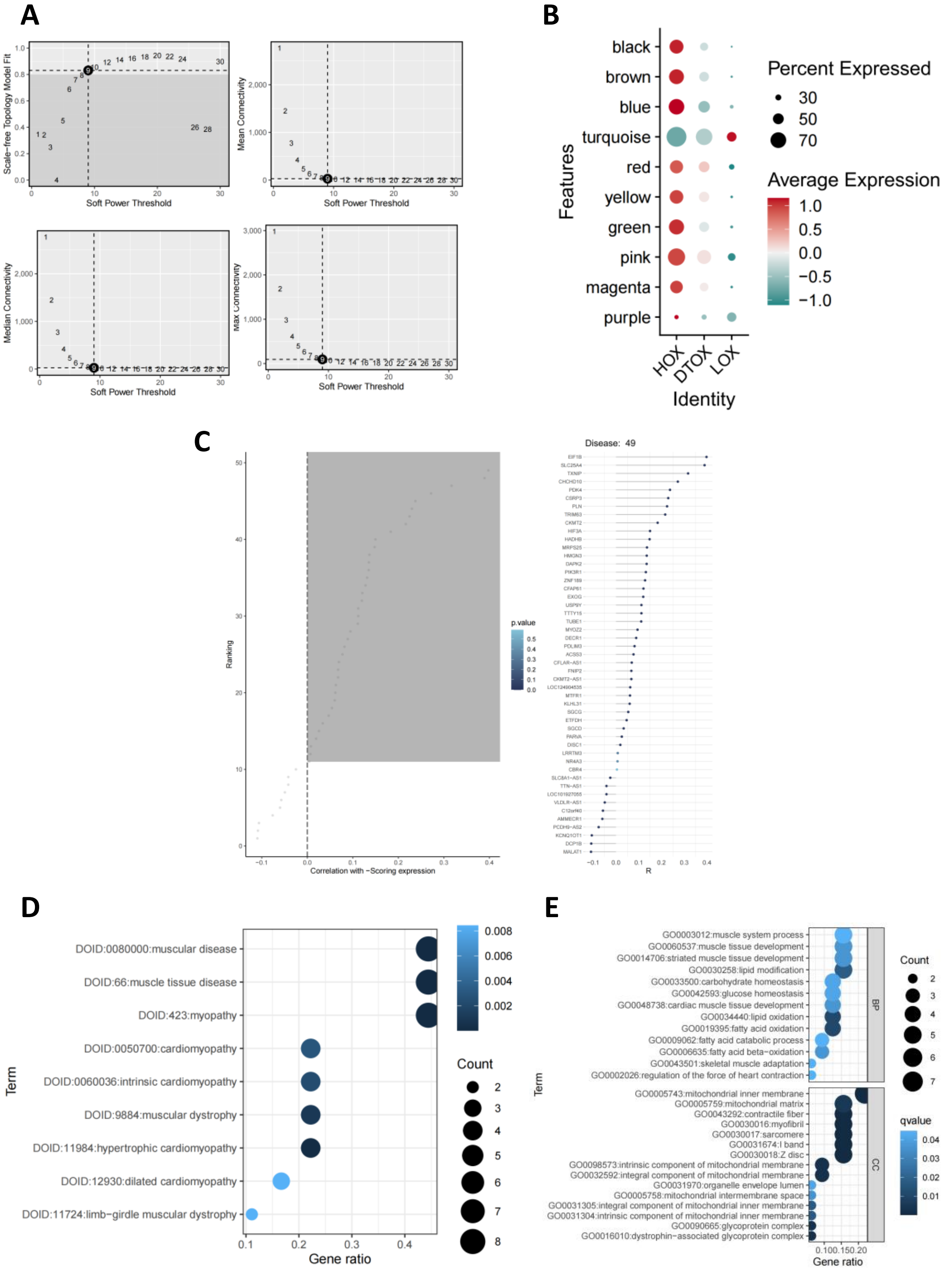

Supplementary Figure S4 Functional Enrichment Analysis of the HOX Core Gene Module. (A) Soft power threshold selection for network construction. The plots show the relationships between the soft power threshold and the scale-free topology model fit and the mean, median, and maximum connectivity. The optimal soft power threshold is chosen based

on the model fit and connectivity. (D) Dot plot showing each gene module's expression level and percentage in HOX, DTOX, and LOX samples. The size of the dots represents the percentage of samples expressing each module, while the colour represents the average expression level. (G) Bar plot showing the correlation of gene expression with scoring expression in different groups. The genes are ranked by their correlation with scoring expression, with colour reflecting the significance of the correlation. The results of correlation analysis with OS. (H) The results of DO analysis of the OS genes. (I) The results of GO analysis of the OS genes.

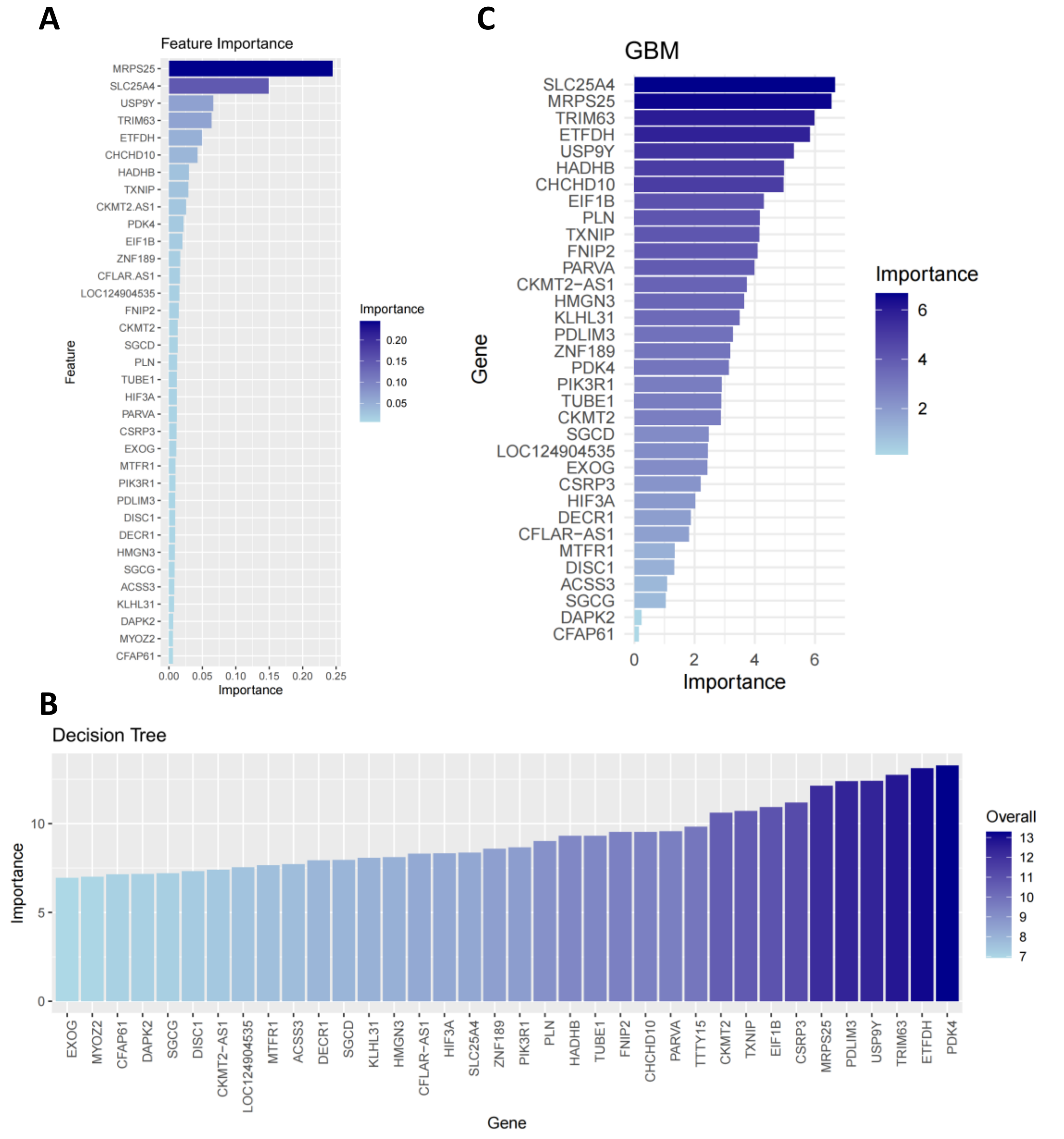

Supplementary Figure S5 Feature Selection Results from Additional Machine Learning Algorithms. (A) Sixteen feature genes were further identified using XGBoost analysis. (B) Ten feature genes were identified using Decision Tree (DT) analysis. (C) Twenty-six feature genes were identified using Gradient Boosting Machine (GBM) analysis.

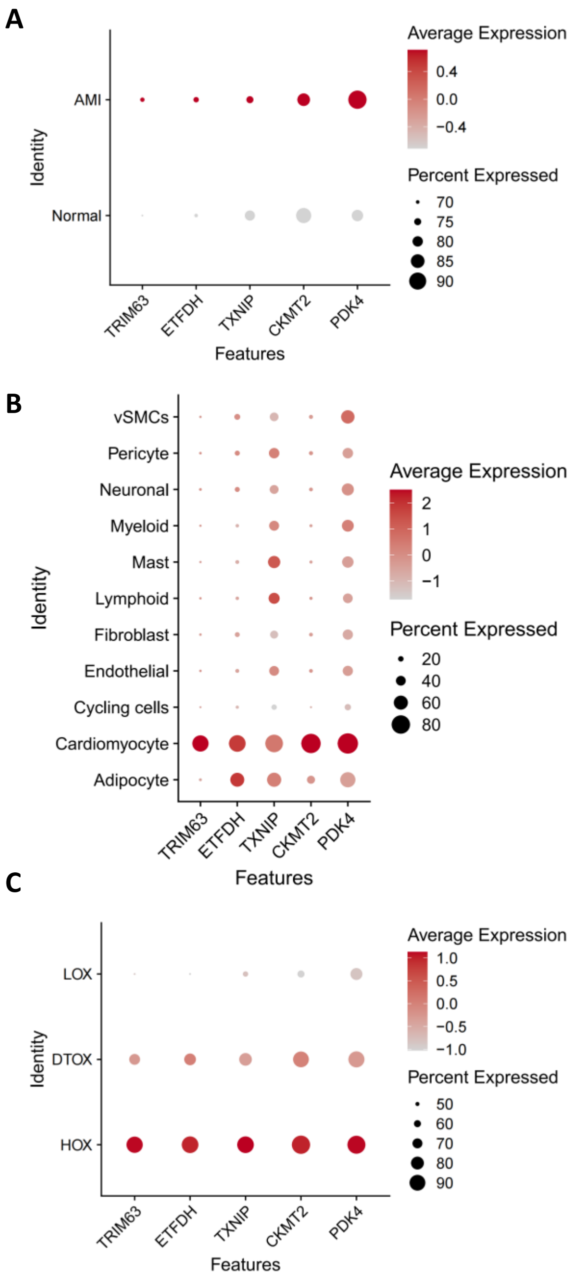

Supplementary Figure S6 Quantitative Expression of Core Feature Genes across Cardiomyocyte Subpopulations. (A) Dot plot showing each feature gene's expression level and percentage across normal and AMI samples. The size of the dots indicates the percentage of samples expressing each gene, while the colour represents the average expression level. (B) Dot plot showing the expression of feature genes (*TRIM63*, *ETFDH*, *TXNIP*, *CKMT2*, and *PDK4*) across different cell types. The size of the dots represents the percentage of cells expressing each gene, and the colour represents the average expression level. (C) Dot plot showing each feature gene's expression level and percentage in cardiomyocytes. The size of the dots indicates the percentage of cells expressing each gene, while the colour represents the average expression level.
